# Low serum Insulin Like Growth Factor - 1 in patients with Stress Urinary Incontinence

**DOI:** 10.1590/S1677-5538.IBJU.2015.0453

**Published:** 2016

**Authors:** Emin Ozbek, Alper Otunctemur, Suleyman Sahin, Levent Ozcan, Murat Dursun, Emrecan Polat, Feti Tulubas, Mustafa Cekmen

**Affiliations:** 1 Department Urology, Okmeydani Training and Research Hospital, Istanbul, Turkey; 2 Department Urology, Derince Training and Research Hospital, Kocaeli, Turkey; 3 Department Urology, Bahcelievler State Hospital, Istanbul, Turkey; 4 Department of Urology, School of Medicine, Istanbul Medipol University, Istanbul, Turkey; 5 Department of Biochemistry, Namik Kemal University Medical Faculty, Tekirdag, Turkey; 6 Department of Medeniyet University Medical Faculty, Istanbul, Turkey

**Keywords:** Urinary Incontinence, Stress, Enzyme-Linked Immunosorbent Assay, Insulin-Like Growth Factor I

## Abstract

**Objective:**

SUI, involuntary loss of urine, occurs when intra abdominal pressure exceeds urethral pressure in women. Recent animal study has shown that there are therapeutic effects of Insulin-like growth factors (IGF-1) on stress urinary incontinence in rats with simulated childbirth trauma. IGF-1 is an important mediator of cell growth, differentiation and transformation in various tissues and stimulates fibroblast proliferation and enhances collagen synthesis. The purpose of the current study was to determine the association between IGF-1 levels and SUI.

**Materials and Methods:**

All patients were evaluated for SUI and divided into two groups: 116 women with SUI and 76 women without SUI. Diagnosis of SUI was based on the International Consultation on Incontinence Questionnaire-Short Form (ICIQSF). Levels of IGF-1 were measured in serum by enzyme-linked immunosorbent assay. The relationship between IGF-1 levels and SUI in patients was evaluated statisticaly.

**Results:**

The mean age of patients wiyh SUI was 49.9±8.6 and 48.7±7.8 in control group. Plasma IGF-1 levels were significantly lower in SUI than in control group (106.5±26.4 and 133.3±37.1ng/mL, respectively, P <0.001). Body mass indexes were higher in women with SUI than women without SUI.

**Conclusion:**

In this study lower serum IGF-1 levels were found to be associated with SUI. Serum IGF-1 level appears to be a specific predictor of SUI, and it may be used in early prediction of SUI in female population.

## INTRODUCTION

Stress urinary incontinence (SUI) is assosiated with high financial, social and emotional costs. SUI affects quality of life as well as sexual function in women (1). SUI is defined as the involuntary loss of urine during increase of abdominal pressure in the absence of bladder contractions; it is the most common type of urinary incontinence in women older than middle age (2). SUI is the most common form of urinary incontinence, occurring in pure or mixed forms in nearly 80% of women with incontinence, according to two European studies (3, 4). One out of every three women will experience SUI at some point in their life. Too many of them “just live with” the condition, too embarrassed to seek help or thinking that it is a “normal” part of aging and having children. The pathogenesis of SUI is thought to be the result of urethral hypermobility secondary to a weakening or disruption of the pelvic floor musculature and/or pubourethral ligament, with a subsequent loss of pressure transmission from the bladder to the urethra upon provocation (5, 6). Previous studies have discovered that numerous factors, including age, obesity, diabetes mellitus, hypertension, menopausal status, parity, pregnancy, psychological factors, and the physical health status of women, could affect their chances of having SUI (7).

Insulin-like growth factors-1 (IGF-1), a peptide hormone that is structurally related to insulin and synthesized by almost all tissues, is an important mediator of cell growth, differentiation and transformation in various tissues (8). IGF-I is a potent mitogen and inhibitor of apoptosis for cell types and exerts all of its known physiologic effects by binding to the IGF receptor (IGF-1R) (9). IGF binding activates IGF-IR, which in turn phosphorylates phosphatidylinositol 3-kinase (PI-3K) and Ras/Raf/mitogen-activated protein kinase (MAPK). Ras/Raf/MAPK and PI-3K play important roles in IGFIR-induced cellular proliferation and inhibition of apoptosis (10). IGF-1 has been shown to stimulate fibroblast proliferation and enhances collagen synthesis (11). IGF-1 also accelerates the growth and differentiation of striated muscle precursor cells in the human urethral sphincter (12). However, to our knowledge the role of IGF-1 has not been explored yet in humans with SUI.

Because of the wide range of their biologic effects and their therapeutic potential, the IGFs have become the focus of research by an increasing number of investigators. The aim of this study was to determine whether any relationship exists between SUI and the level of IGF-1.

## MATERIAL AND METHODS

We performed a prospective cross-sectional study of participants who visited Okmeydani Training and Research Hospital from February 2011 to January 2013. All women were evaluated for SUI and menopausal period. The women were divided into two groups: 116 women with SUI and 76 women without SUI (control). Postmenopausal status was defined as the cessation of menses for at least 1 year, and perimenopausal status as skipped menstruation with perimenopausal symptoms. Premenopausal women who have regular menses were assessed. 65 women were postmenopausal and 51 women were premenopausal in study group. Also, 40 women were postmenopausal and 36 women were premenopausal in control group. We used the International Consultation on Incontinence Questionnaire-Short Form (ICIQ-SF) to evaluate SUI and the question: “During the past 12 months, have you leaked or lost control of even a small amount of urine with activity like coughing, lifting, or exercise?” defined stress UI. Also, we assessed the women for SUI at gynecologic position and we determined cough stress test. In the calculations, contiinence was defined as no incontinence at all. The urodynamic study was used in patients with symptoms of mixed urinary incontinence and in cases of clinically suspicious diagnosis. Exclusion criteria were: urge incontinence, mixed urinary incontinence with dominant urge component, intrinsic sphincter deficiency, neurogenic bladder (multiple sclerosis, meningomyelocele, spinal cord injury), genital surgery history, severe mental illness and severe physical handicap. Patients who were pregnant were excluded.

IGF-1 was quantified via an enzyme-linked immunosorbent assay (ELISA) using the Immulite Analyzer kit (Diagnostic Products Corp., Caernarfon, Gwynedd, UK).

### Statistical analysis

Statistical analyses were performed by the Statistical Package for Social Sciences, version 15.0, software (SPSS Inc., Chicago, IL, USA). The quantitative demographic values were evaluated by student's t or Mann Whitney U tests whether the parameters were suitable for normal distribution or not. If the parameters were qualitative chi-square test was used. Spearman correlation test was performed to analyse the association between SUI and IGF-1 level. All tests were performed using a 2-tailed analysis, and a P value of <0.05 was considered statistically significant.

## RESULTS

**Figure 1:**
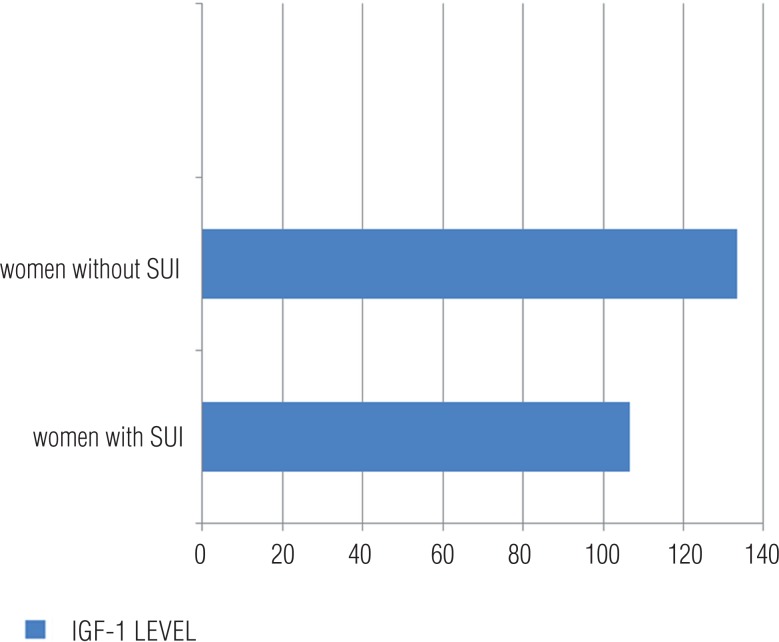
Serum IGF-1 levels between study and control group. The distribution of IGF-1 levels between study and control group(106.5± 26.4 ng/mL women with SUI; 133.3± 37.1 women without SUI respectively, P < 0.001).

192 women were included in the study and were divided into two groups: 116 women with SUI and 76 women without SUI (control). The baseline characteristic properties of study patients are demonstrated in Table-1. The mean age was 49.9±8.6 in women with SUI and 48.7±7.8 in women without SUI; no statistical difference were found for age between groups. There was no statistical difference between groups for weight, height, parity. Number of parity was 3.1±1.4 in patients with SUI and 2.9±1.1 in patients without SUI. Body mass indexes (BMI) were higher in women with SUI than women without SUI. There was statistically difference for BMI between women with SUI and control group (p<0.001). Also DM was higher in women with SUI than control group but there was no difference for DM between groups (p=0.15). We compared the IGF-1 levels between groups. Our results showed that women with SUI have lower IGF-1 levels than control group (Figure-1). Mean IGF-1 level was 106.5±26.4 in women with SUI and 133.3±37.1 in control goup, respectively. There was statistically difference for IGF-1 levels between women with SUI and control group (p<0.001)). The IGF-1 levels were negatively correlated with ICIQ-SF score (r=-0.583, P <0.01).

**Table 1:** Characteristics of women with or without SUI.

	Women with SUI (n=116)	Women without SUI (n=76)	P value
Age (mean±sd)	49.9 ± 8.6	48.7 ± 7.8	0.08
Height(cm, mean±sd)	158 ± 5.7	160 ± 4.9	0.18
Weight(kg, mean±sd)	74.6 ± 13.1	73.2 ± 12.3	0.1
BMI (mean±sd)	29.8 ± 5.7	27.3 ± 4.3	< 0.001*
Parity (mean±sd)	3.1 ± 1.4	2.9 ± 1.1	0.06
DM(%)	29	25	0.15
IGF-1 (ng/mL, mean±sd)	106.5 ± 26.4	133.3 ± 37.1	< 0.001*

* P < 0.05 was accepted as statistically significant.

**BMI** = Body mass index; **DM** = Diabetes mellitus; **IGF-1** = Insulin like growth factor-1

## DISCUSSION

The purpose of this investigation was to examine the association between IGF-1 levels and SUI in women. The results of this study showed that the patients with SUI had significantly lower IGF-1 levels than control group. Moreover, our study revealed that there was a negative correlation between IGF-1 levels and ICIQ-SF score. To our knowledge, this is the first study that shows the serum levels of IGF-1 in SUI patients. This study suggested that IGF-1 might contribute to the pathophysiology of SUI in patients. In a previous study, IGF-1 levels were lower in diabetic women (13). Also, we found that IGF-1 levels were lower in women with SUI; however in our series, DM prevalence was similar between women with and without SUI. In this context, IGF-1 levels were lower in women with SUI independently of DM.

An effective closure of the female urethra in stress situations depends on an integrated action of various anatomical intra and extraurethral structures. The most important extraurethral structures are the suburethral vaginal wall, the pubourethral ligaments, the pubococcygeus muscles, and the periurethral connective tissue. Fibrous connective tissue is mainly composed of collagen and structural glycoproteins and forms an important part of the supportive structures of the genitourinary region (14). Female urinary incontinence is a common problem that disables many women, especially after menopause. Recent research has focused on functional changes on the pelvic floor and the condition of the fibrous connective tissue, which is their main constituent as factors that play a significant role in the development of SUI (15).

The mechanical and supportive properties of the pelvic connective tissue are determined by the structure of the main molecules that constitute the tissue (collagen, elastin, proteoglycans and glycoproteins), their interactions with each other and their overall proportions. Alterations in the quantity and organization of collagen fibers significantly affect the tensile strength of the endopelvic fascia and consequently determine an attenuation in the support that provide to the bladder neck and bladder base, resulting in urethral hypermobility that causes 80–90% of SUI. Menopause and aging serve as important factors for the onset of SUI, also for the mechanism for how they affect the metabolic processes within the connective tissue (16). The main constituent of the connective tissue in the ligaments and the suburethral-vaginal wall is collagen. Collagens of type I and type III are the predominant components in this kind of connective tissue and are responsible for the tensile strength of the tissue (17). Several studies have been reported that total collagen reduction of the paravaginal fascia is associated with the development of SUI. In several papers, in incontinent women a decreased expression in collagen type I or in collagen III has been demonstrated. For example, Ulmsten and Ekman (18) showed that collagen content in biopsies from skin and ligamentum rotundum of women with a long history of stress incontinence, compared with that of continent controls, was 25-40% less than that of continent women. Also Chen et al. (19) demonstrated 60% less collagen content in the vaginal wall of women with SUI compared to age-matched continent women. Falconer et al. (20) suggested that women with SUI have an altered connective tissue metabolism causing decreased collagen production. Liapis et al. (14) have shown a significant reduction amount of type I collagen in about 53% patients with SUI. Kean et al. (21) showed that the nulliparous women with SUI had significantly less collagen in their tissues compared with the continent controls. In addition, they demonstrated a decreased ratio of type I to type III collagen in women with SUI. In relation to collagen type III content Bergman et al. (22) showed that it was significantly reduced in specimens from patients with SUI.

Metabolic processes that occur within the connective tissue seem to play an important role in regulating collagen concentrations in the periurethral region (3). Several factors, such as age, mechanical stress, hormones, enzymes and their inhibitors, growth factors and cytokines, are involved in these processes (19). Collagen production is primarily regulated by fibroblasts under the influence of specific growth factors such as IGF-1. Previous studies have shown that IGF-I is essential for collagen production by fibroblasts (23). The activation of the MAPK signalling pathway in connective tissue by autocrine production of IGF-1 is essential for collagen expression. In a previous study, the inhibition of IGF-1 signalling pathway such as Akt-1, MAPK and c-Raf may lead to a decrease in fibroblast proliferation and subsequent reduction in collagen deposition (24). In our study, IGF-1 levels were significantly lower in patients with SUI compared to control group (p <0.001). Higher IGF-1 levels may prevent SUI by increasing collagen production. Also, hormonal changes in reproductive period may affect collagen production such as estrogen. The reduction of estrogen levels in postmenopausal period contributes to the development of SUI also affecting collagen content and metabolism in the supportive pelvic connective tissue (16). On the other hand, about 44% of the study group consists of premenopausal patients. This finding can define that IGF-1 level is more effective than estrogen level on collagen production.

Therapeutic effects of IGF-1 on SUI have been studied in animal experiments in a recent study. IGF-1 treatment showed significant improvement in leak point pressure, urethral baseline pressure and urethral responses. IGF-1 treatment increased Akt phosphorylation and induced cellular proliferation and antiapoptotic effects in the urethral tissue. IGF-1 treatment may accelerate recovery from SUI in rats (25). In English literature there is no clinical study concerning the role of serum IGF-1 in SUI patients. To the best of our knowledge this is the first clinical study with humans in literature.

There are some limitations in our study. Small sample size was the major limitation in this study.

In conclusion, this study was conducted to determine a relationship between serum IGF-1 level and SUI. Serum IGF-1 level appears to be a specific predictor of SUI, and it may be used in early prediction of SUI in female population. Furthermore, molecular medical treatment of SUI with IGF-1 may be possible and effective. However, further large scale clinical and molecular studies are needed to confirm the pathophysiological role of IGF-1 in SUI patients.
